# Dynamics of Spinodal Decomposition in a Ternary Gelling System

**DOI:** 10.3390/gels4020026

**Published:** 2018-03-22

**Authors:** Yutaro Yamashita, Miho Yanagisawa, Masayuki Tokita

**Affiliations:** Department of Physics, Graduate School of Science, Kyushu University, 744 Motooka, Fukuoka 810-0935, Japan; yama_cf@yahoo.co.jp (Y.Y.); myanagi@cc.tuat.ac.jp (M.Y.)

**Keywords:** sol-gel transition, site-bond correlated-percolation model for polymer gelation, gelation temperature, cloud point temperature, spinodal temperature, spinodal decomposition

## Abstract

The phase diagram and phase transitions of the ternary system of gelatin, water and poly(ethylene glycol) oligomers were studied as a function of the weight fraction of gelatin and the weight fraction and molecular weight of poly(ethylene glycol) oligomers. It was found that both phase separation and the sol-gel transition occur in this ternary system. The relative position of the phase separation line and the sol-gel transition line depends on the weight fraction and the molecular weight of the poly(ethylene glycol) oligomer that coexists in the solution. All aspects of the phase diagram are sensitive to the molecular weight of the poly(ethylene glycol) oligomer. Since the phase separation line crosses the sol-gel transition line in the phase space that is created by the temperature and the weight fraction of gelatin, the phase space is typically divided into four regions, where each region corresponds to a definite phase. The transitions between mutual phases were studied using the light-scattering technique.

## 1. Introduction

There are many water-soluble polymers distributed throughout the natural world. Some of these can be synthesized artificially, while others solely originate from biological systems. Since water-soluble polymers are environmentally sustainable, they are widely used in science, technology and even in our day-to-day lives. The phase behavior and the phase transition of polymer solution play important roles in the fabrication processes of polymeric materials. Therefore, it may be important to obtain information on the phase structure of the water-soluble polymer in the solution state for the effective fabrication of many practical commodities. In this paper, we report on the phase behaviors of gelatin, water and poly(ethylene glycol) (PEG) in a system.

Gelatin is a typical protein derived from collagen in the skin and bones of animals. It is well known that the aqueous solution of gelatin shows sol-gel transition. The gel of gelatin is widely used in food, and hence, the sol-gel transition of gelatin solution has been studied in detail thus far [1,2,3]. On the other hand, PEG is also well known as a neutral water-soluble polymer. The polymer and its oligomers interact with proteins, and hence, they are used as precipitating agents of protein in protein crystallography. The phase diagram of the aqueous PEG system has been also studied [4]. It is clarified that the aqueous PEG solution shows the phase separation only at very higher temperature regions rather than the room temperature region.

It has been reported that the phase separation becomes obvious when methanol is added to the aqueous solution of gelatin as the third component of the system [5]. Although the results are not systematic, it was also reported that the aqueous gelatin solution shows phase separation when a trace amount of large-molecular-weight PEG is added to the solution [6]. Therefore, the ternary system of the aqueous gelatin with PEG has attracted much attention since these studies, and then, many more studies have been done, especially in pharmaceutical science [7,8,9,10,11]. However, the systematic study of the phase behaviors of the ternary system of gelatin, water and PEG has yet to be done.

Here, we report on the systematic study of the phase behaviors in the ternary system of gelatin, water and PEG. A typical phase diagram of the system has been described in the previous report [12]. The key point of the previous study is the choice of PEG. We choose the oligomers of PEG with various molecular weights in this study. As such, the weight fraction of PEG oligomer can be changed widely, and hence, the overall aspects of the phase behaviors of the ternary system of gelatin, water and PEG were revealed. In this study, the concentration range of PEG in the system was changed widely to obtain the phase diagram of the system in the entire parameter space. The phase separation of the solution was observed in addition to the sol-gel transition of the solution in the ternary system. It was found that two phase transition lines of the phase separation and the sol-gel transition cross in the $T-\varphi_{g}$ phase space, where *T* and $\varphi_{g}$ represent the temperature and the weight fraction of gelatin, respectively. Therefore, the phase space of the ternary system is typically divided into four regions corresponding to the definite phases. The phase transitions were investigated mainly by the microscopic observations in the previous study. Thus, the light-scattering measurements were taken to clarify the characteristics of the phase transitions between these four phases in this study.

## 2. Results

### 2.1. Phase Diagram

**Figure 1:**
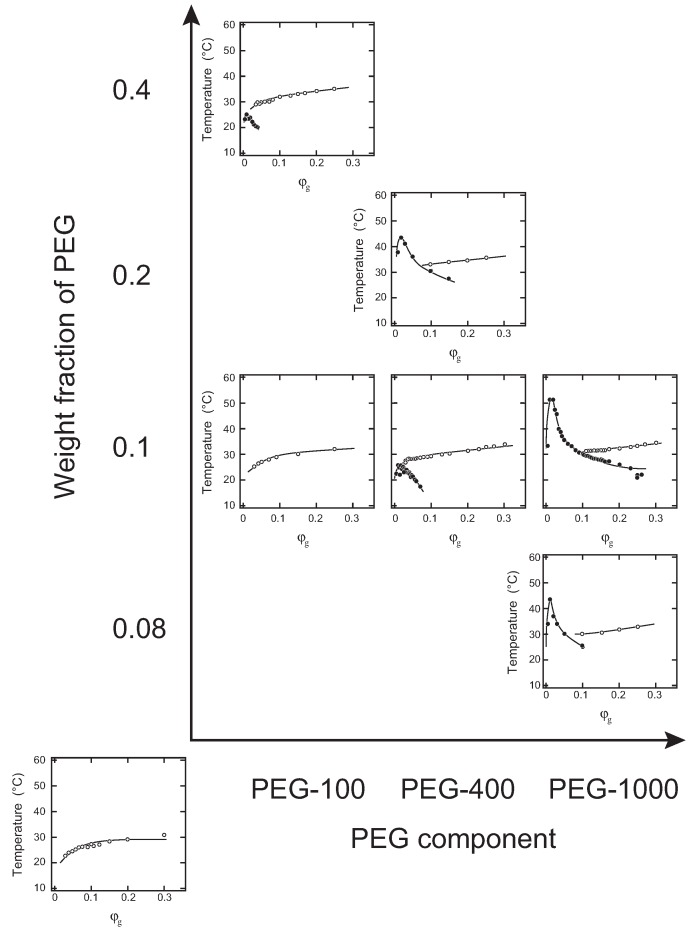
The phase diagrams of systems with respect to gelatin, water and oligomers of poly(ethylene glycol) (PEG). The cloud point (closed circles) and the gelation temperature (open circles) are plotted as a function of the weight fraction of gelatin, $\varphi_{g}$. Each phase diagram is aligned as a function of the weight fraction of PEG and the molecular weight of PEG. The phase diagram of the aqueous gelatin solution is shown in the bottom left corner. The series of phase diagrams at a weight fraction of 0.1 is a reproduction from a previous report [12]. PEG-100: diethylene glycol; PEG-400: PEG at M. W. ∼400; PEG-1000: PEG at M. W. ∼1000.

In Figure 1, we show the phase diagrams of the system that are determined under various conditions of the weight fraction of gelatin, as well as the weight fraction and the molecular weight of the oligomer PEG. The cloud point and the gelation temperature are plotted as a function of the weight fraction of gelatin in each phase diagram. The phase diagram itself is arranged by two parameters of the weight fraction and the molecular weight of the oligomer PEG.

It is clear from the phase diagram that the aqueous gelatin solution only shows the sol-gel transition, as can be seen in the bottom left corner. The phase diagram of the aqueous gelatin solution is similar to those seen in previous reports [2,5]. In the phase diagrams of diethylene glycol (PEG-100) systems, it was found that the solution only shows sol-gel transition when the weight fraction of PEG-100 is 0.1. Although data are not shown, the same phase diagrams were obtained in the systems of triethylene glycol (PEG-150) and tetraethylene glycol (PEG-200) at a weight fraction of 0.1 [12]. In contrast, the phase separation became obvious when the weight fraction of PEG-100 was increased to 0.4. The results indicate that the solution of PEG-100 is a poor solvent for gelatin when the weight fraction is increased to 0.4 because the phase separation of the polymer solution is a characteristic behavior that occurs in poor solvent systems [13].

It was also found from the series of the phase diagrams with the weight fraction of PEG in the 0.1 system that the phase separation line appears when the molecular weight of the PEG oligomer is increased at a constant weight fraction. In addition to this, the critical temperature is raised and is shifted to the lower concentration region of gelatin with the molecular weight of the PEG oligomer that coexists in the system. The phase diagrams shown in Figure 1 indicate that the relative position of the phase separation line and the sol-gel line varies with the weight fraction and the molecular weight of the PEG oligomer. It was found that the phase diagrams of PEG-100 at a weight fraction of 0.4 and that of PEG-400 at a weight fraction of 0.1 in the system become similar. A similarity in the phase diagrams was also observed between PEG-400 at a weight fraction of 0.2 and PEG-1000 at a weight fraction of 0.08 in the system. The results indicate that the phase diagram of the ternary system of gelatin, water and PEG can be controlled by the appropriate choice of the molecular weight and the weight fraction of PEG oligomer.

**Figure 2:**
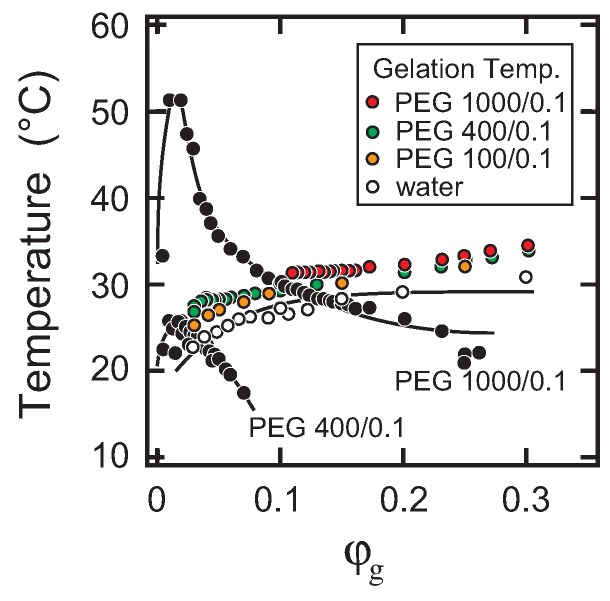
The phase diagrams in the series of PEG oligomers at a weight fraction of 0.1. Sol-gel transition temperatures are expressed by colored circles, and the phase separation temperatures are expressed by solid (black) circles, respectively. The open circles (white) represent the sol-gel transition line of the aqueous gelatin solution.

Among others, the series of the phase diagrams at a PEG weight fraction of 0.1 clearly shows the effects of the molecular weight on the phase diagram. Phase diagrams of the system at a weight fraction of PEG oligomers of 0.1 (PEG-100, PEG-400, PEG-1000 systems) in Figure 1 are summarized as Figure 2 with the phase diagram of the aqueous gelatin solution, where phase separation temperatures and sol-gel transition temperatures are shown in closed circles and colored circles, respectively. Only sol-gel transition is observed in the system of PEG-100 at a weight fraction of 0.1, while both the sol-gel transition and the phase separation are observed in the systems of PEG-400 (M. W. ∼ 400) and PEG-1000 (M. W. ∼ 1000) at the same weight fraction of PEG. The appearance of the phase diagram is completely distinct from that of the PEG-100 system. Since the weight fraction of the oligomer in the solution is the same, the number of oligomers in the solution of PEG-1000 is only about one-tenth of that of the PEG-100 system. The results, therefore, indicate that the molecular weight of the PEG oligomer is very effective at changing the phase separation line rather than changing the sol-gel transition line. As the PEG chain becomes longer, the phase separation temperature increases dramatically. In contrast, the positions of the sol-gel transition lines are almost independent of the molecular weight of PEG under the present experimental conditions.

### 2.2. Light Scattering

**Figure 3:**
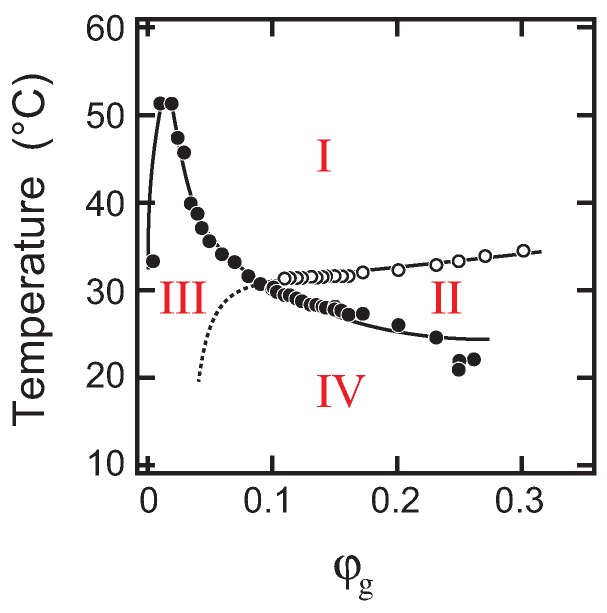
The phase diagram of the PEG-1000 system at the weight fraction of 0.1 in Figure 1. The $T-\varphi_{g}$ phase space is divided into four regions. Region I: one-phase sol; Region II: one-phase gel; Region III: two-phase sol; Region IV: two-phase gel. The presence of the phase boundary between two phases of III and IV is not obvious in the present study.

When the phase structure of the system was revealed, we were interested in how the transition between phases occurred. In the phase diagram in Figure 1, the systems of PEG-100 at a weight fraction of 0.4 and PEG-400 at a weight fraction of 0.1 are too delicate to study the transition behaviors because the sol-gel transition line terminates near the critical point of the phase separation line. Such a point is physically interesting to study, but difficult to analyze, and hence, we have left it for future studies. Accordingly, we choose the system of PEG-1000 at a weight fraction of 0.1 to obtain the fundamental characteristics of the phase transition between phases. Since two phase boundaries cross in this system, the $T-\varphi_{g}$ phase space is divided into four regions, I, II, III and IV, as shown in Figure 3. Each region corresponds to the definite phase as follows.

- Region I ⟶ one-phase sol
- Region II ⟶ one-phase gel (transparent)
- Region III ⟶ two-phase sol
- Region IV ⟶ two-phase gel (opaque)

Among the above regions, Regions I and II correspond to the simple sol and the simple gel phases so far observed in the aqueous gelatin solution. The difference between the gel formed in Regions II and IV is clear in appearance, but the definition of the phase separation line in the gel state is not clear enough. Finally, the presence of the phase boundary between III and IV is not clarified in the present study.

**Figure 4:**
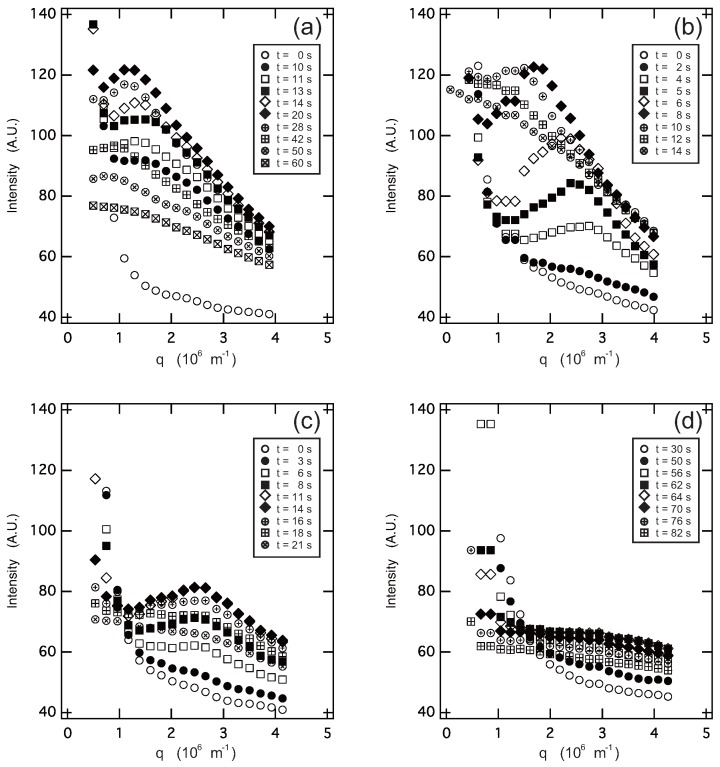
The light-scattering profile from the system undergoing phase transition. The relative intensity of the scattered light from the sample is plotted as a function of the scattering vector, *q*. The series of the scattering profiles is plotted as a function of the elapsed time after the temperature change, which is given in each figure. The systems are $\varphi_{g}=0.05$ (*T*; 40.0 → 10.0 ${}^{\circ }$C) (**a**); $\varphi_{g}=0.1$ (*T*; 38.0 → 15.0 ${}^{\circ }$C) (**b**); $\varphi_{g}=0.2$ (*T*; 60.0 → 15.0 ${}^{\circ }$C, single quench) (**c**); and $\varphi_{g}=0.2$ (*T*; 30.0 → 15.0 ${}^{\circ }$C) (second quench of the double quench) (**d**). The weight fraction of PEG-1000 is fixed at 0.1.

The light-scattering measurements are made under the following quench conditions, and the results are given in Figure 4.

1. $\varphi_{g}=0.05$, *T* is changed from 40.0 to 10.0 ${}^{\circ }$C by single quench (Region I → Region III).
2. $\varphi_{g}=0.1$, *T* is changed from 38.0 to 15.0 ${}^{\circ }$C by single quench (Region I → Region IV).
3. $\varphi_{g}=0.2$, *T* is changed from 60.0 to 15.0 ${}^{\circ }$C by single quench (Region I → (Region II) → Region IV).
4. $\varphi_{g}=0.2$, *T* is changed from 60.0 to 30.0, then to 15.0 ${}^{\circ }$C by double quench (Region I → Region II → Region IV).

**Figure 5:**
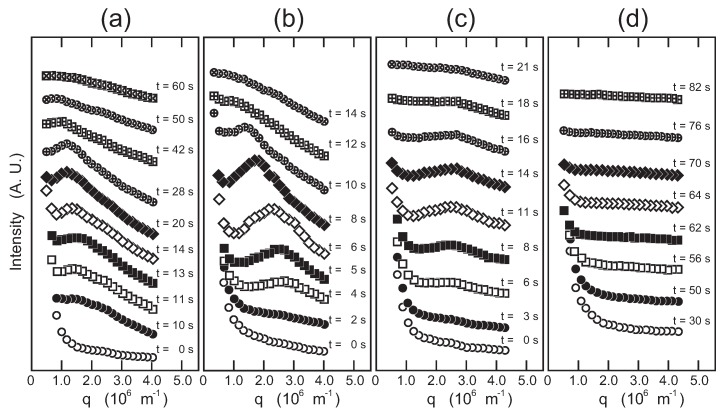
The scattering profiles shown in Figure 4 are vertically shifted with respect to each other to show the time evolution of the maximum that appeared in the scattering profile. Symbols are the same as used in Figure 4. The systems are $\varphi_{g}=0.05$ (*T*; 40.0 → 10.0 ${}^{\circ }$C) (**a**); $\varphi_{g}=0.1$ (*T*; 38.0 → 15.0 ${}^{\circ }$C) (**b**); $\varphi_{g}=0.2$ (*T*; 60.0 → 15.0 ${}^{\circ }$C, single quench) (**c**); and $\varphi_{g}=0.2$ (*T*; 30.0 → 15.0 ${}^{\circ }$C) (second quench of the double quench) (**d**). The weight fraction of PEG-1000 is fixed at 0.1.

Since the scattering profiles shown in Figure 4 are strongly overlapped, the scattering profiles are shifted vertically and shown in Figure 5 for the sake of clarity.

#### 2.2.1. System $\varphi_{g}=0.05$

It is expected from Figure 3 that the transition that occurs during this process is simple phase separation from the one-phase sol (Region I) to the two-phase sol (Region III). The sol-gel transition of the system may not have significant effects on the phase separation since the phase separation occurs in a rather higher temperature region of about 35 ${}^{\circ }$C. The in situ optical microscope observation indicates that the phase separation proceeds by the nucleation-growth process in the slow cooling process of the solution [12]. In contrast, the light-scattering results from the quenched solution, Figure 4a and Figure 5a, show the characteristic behaviors of the spinodal decomposition. Namely, the scattering intensity shows a maximum after 10 s from the quench of the solution. The magnitude of the scattering vector at which the scattering intensity shows the maximum, $q_{\max}$, is about $q_{\max}∼2\times 10^{6}\;$m${}^{-1}$. The position of the maximum in the scattering profile moves towards the smaller values of *q* with time, and then, it becomes invisible to the scattering profile after 60 s from the quench of the solution. The results strongly suggest the emergence and divergence of the density fluctuations. These results are similar to the previous studies that were made in different systems [14,15,16,17]. It was, therefore, found that the phase separation of the solution occurs by spinodal decomposition when the solution is quenched from Region I to III. Taking into account the results of the previous in situ optical microscope observation, the phase transition observed here is the simple phase separation.

#### 2.2.2. System $\varphi_{g}=0.1$

In this system, the light-scattering results, Figure 4b and Figure 5b, showed the similar behaviors with the system $\varphi_{g}=0.05$. However, it is clear from Figure 4b and Figure 5b that the maximum in the scattering profile appears at much earlier time frames than that in the system $\varphi_{g}=0.05$: the scattering profile shows a maximum at about $q_{\max}∼3\times 10^{6}\;$m${}^{-1}$ after 2 s from quenching. The results suggest that the spinodal decomposition is considerably accelerated in the $\varphi_{g}=0.1$ system. The quench process of the system is special because the system is cooling through the cross-over point of the sol-gel transition line and the phase separation line. Therefore, both the phase separation and the network formation by the sol-gel transition may equally effect the time evolution of the system.

#### 2.2.3. System $\varphi_{g}=0.2$ in Single Quench

The scattering results of this system, Figure 4c and Figure 5c, were totally altered from the previous two systems. The maximum appeared in the scattering profile, but it was observable only in a certain time interval from 6 to 18 s after the quenching of the solution. Besides, the magnitude of the scattering vector at the maximum, $q_{\max}∼2.5\times 10^{6}\;$m${}^{-1}$, was time independent throughout the observation. The final state of the system is the two-phase gel, which is opaque in appearance. A similar phenomenon was observed in the gelation process of agarose [18].

#### 2.2.4. System $\varphi_{g}=0.2$ in Double Quench

**Figure 6:**
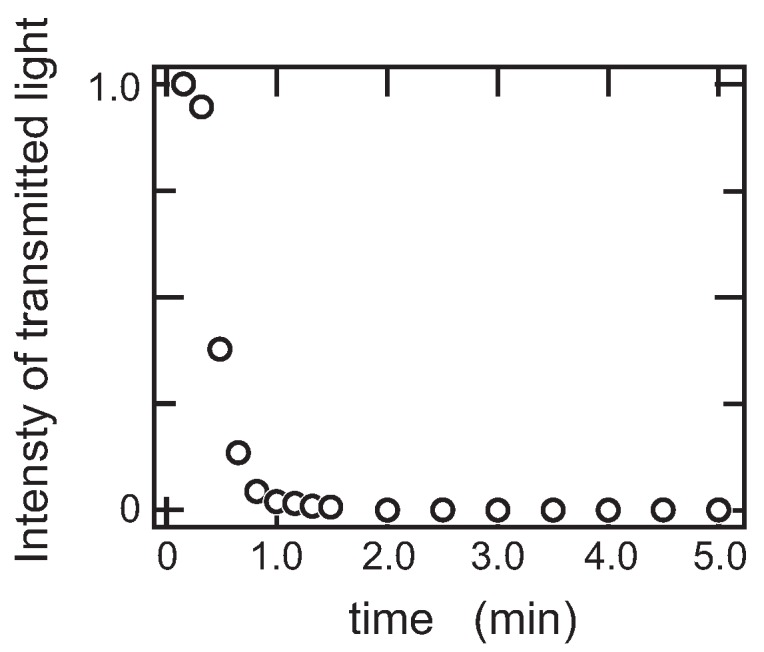
The time evolution of the relative intensity of the transmitted light after the second quench of the system $\varphi_{g}=0.2$ from 30.0 to 15.0 ${}^{\circ }$C. The weight fraction of PEG-1000 is 0.1.

The phase transition behavior of this system was studied by the double quench process to reveal the transition behavior of this system in detail. In the double quench process, the solution was firstly quenched into 30.0 ${}^{\circ }$C from the solution state at 60.0 ${}^{\circ }$C where the one-phase gel, which is transparent in appearance, was formed: Region II in Figure 3. The solution was kept at 30.0 ${}^{\circ }$C overnight to ensure the gelation at this temperature. Then, the transparent gel thus obtained was quenched into the phase separation region at 15.0 ${}^{\circ }$C, and the light-scattering profiles were gained. The scattering results, Figure 4d and Figure 5d, did not show any singular behaviors. Only the opaqueness of the gel is increased after the second quench process. The results of the turbidity measurements after the second quench are given in Figure 6 to complement the light-scattering results. It is clear from Figure 6 that the opaqueness of the gel increases immediately after the quenching of the transparent gel from 30.0 to 15.0 ${}^{\circ }$C. The second quench process of the system corresponds to the transition from the homogeneous one-phase gel to the inhomogeneous two-phase gel.

## 3. Discussion

In this study, the interaction between gelatin and PEG was viewed in the form of a phase diagram of the ternary system. It was found from the phase diagrams that the two transitions of the phase separation and the sol-gel transition appear in the $T-\varphi_{g}$ phase space in the ternary system of gelatin, water and PEG oligomer. The relative position of the phase separation line and the sol-gel transition line was found to depend both on the concentration and the molecular weight of PEG oligomer. The results indicate that the addition of PEG into the aqueous solution of gelatin deteriorates the quality of the solvent as has been observed in the ternary system of gelatin, water and methanol [5]. The phase behaviors of the present ternary system that are shown in Figure 1 are essentially in agreement with the site-bond correlated-percolation theory of the polymer gelation [19,20].

**Figure 7:**
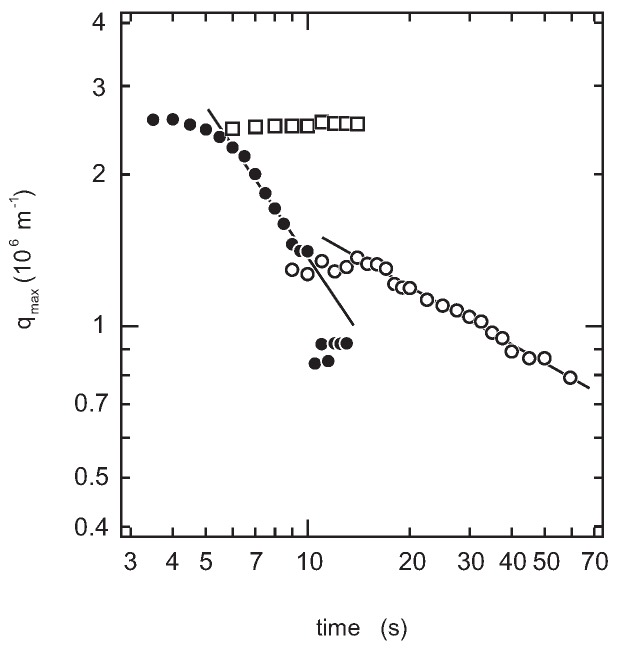
The time evolution of $q_{\max}$. Results are shown for the $\varphi_{g}=0.05$ system (open circles), the $\varphi_{g}=0.1$ system (closed circles) and the $\varphi_{g}=0.2$ system with single quench (open squares). The weight fraction of PEG-1000 is fixed at 0.1. The slopes of the lines drawn for the $\varphi_{g}=0.05$ system are −0.4 and for the $\varphi_{g}=0.1$ system, −1.

The time evolution of the light-scattering profiles from the solution shown in Figure 4 and Figure 5 indicates that the spinodal decomposition plays essential roles in the quench processes of the system. Therefore, the time evolution of the magnitude of the scattering vector at the maximum that appears in the scattering profile, $q_{\max}$, is shown in Figure 7. The reciprocal of $q_{\max}$ is the measure of the correlation length, $\xi_{\mathrm{SD}}$, over which the density fluctuation of the spinodal decomposition correlates. The least squares analysis of the results shown in Figure 7 yields the following.

(1) $$\begin{array}{cc} q_{\max}\propto t^{-0.4} & \;\left(\varphi_{g}=0.05,15\;s\leq t\right) \end{array}$$

(2) $$\begin{array}{cc} q_{\max}\propto t^{-1} & \;\left(\varphi_{g}=0.1,6\;s\leq t\right) \end{array}$$

(3) $$\begin{array}{cc} q_{\max}\propto t^{0} & \;\left(\varphi_{g}=0.2,3\;s\leq t\leq 18\;s\right) \end{array}$$

The late stage behavior of the $\varphi_{g}=0.05$ system is close to the typical behavior of the simple liquid-liquid phase separation, $q_{\max}∼t^{-\alpha }$, where α=1/3 [21]. Since the sol-gel transition temperature is quite similar to that of aqueous gelatin solution without PEG, as shown in Figure 2, the PEG addition might not have affected the gelation of gelatin via triple-helix formation at this concentration of PEG oligomer. In contrast to the dilute system, it is clearly observed that the phase separation is much accelerated in the dense system of φ=0.1, where α≃1. In this accelerated phase separation, the following two factors are considered: (i) the attractive interaction due to the gelation and (ii) viscoelastic phase separation. Such acceleration of the phase separation has been studied theoretically, as well as experimentally so far [22,23,24,25,26,27]. Since the sol-gel transition temperature was slightly above 30 ${}^{\circ }$C, entanglement of the polymers might contribute to the elastic nature of the system and bring viscoelastic phase separation. Such viscoelastic phase separation is expected to be observed by adding much longer PEG [6]. Besides, the previous studies suggest that the viscoelastic phase separation is strongly affected by the experimental conditions such as the cooling rate and the shear rate, so detailed and precise experiments are required for the full understanding of the viscoelastic phase separation in this system [28,29,30]. Such studies are now in progress and will be reported elsewhere.

On the other hand, the phase transition in the $\varphi_{g}=0.2$ system is completely different from that of the previous two systems as shown in Figure 4, Figure 5 and Figure 7, $q_{\max}\propto t^{0}$. It is clear from Figure 3 that the phase separation line is buried in the gel phase in this system. Therefore, the quench depth is deeper for the gelation than that of phase separation, and hence, the gelation proceeds faster. The density fluctuation of the spinodal decomposition appears in the system; however, it cannot grow beyond the size of the polymer network of the gel. Then, the density fluctuations are spontaneously pinned into the polymer network of the gel in the intermediate stage. Finally, the density fluctuations once pinned in the polymer network of the gel are smeared out by the cross-linking reaction. The spontaneous pinning of the density fluctuations was reported in the late stage of the spinodal decomposition in the polymer mixture and the gelation process of agarose solution [18,31]. We, then, carried out the double quench experiment to clarify the competition of the gelation and the phase separation in the $\varphi_{g}=0.2$ system. The transparent gel was formed in the first quench process from Region I to II. The double quench process, therefore, corresponds to the case where the gelation reaction proceeds much earlier than the phase separation. It is clear that the time evolution of the light-scattering profile does not show any characteristic behavior, and only the turbidity of the gel increases after the second quench process as shown in Figure 4d, Figure 5d, and Figure 6. Although the polymer network of the gel is formed, the phase separation can occur in the system. This further suggests that the phase separation and the sol-gel transition are independent phenomena under the present experimental conditions. The results are what would be expected from the site-bond correlated-percolation model of the polymer gelation. Since the phase separation occurs in the polymer network of the gel, the phase separation is restricted to the microscopic scale by the polymer network of the gel, which prevents the divergence of the density fluctuations. Such a phase separation has been called microphase separation so far. The microphase separation within the gel is also expected in the equivalent phase diagram of the gelling system [32]. The phase boundary that separates Phases II and IV, which is drawn as the extrapolation of the phase separation line in the lower concentration region in Figure 3, should be recognized as the microphase separation line in the homogeneous gel. The physical meaning of the phase separation line changes at the cross-over point of the sol-gel transition line and the phase separation line.

**Figure 8:**
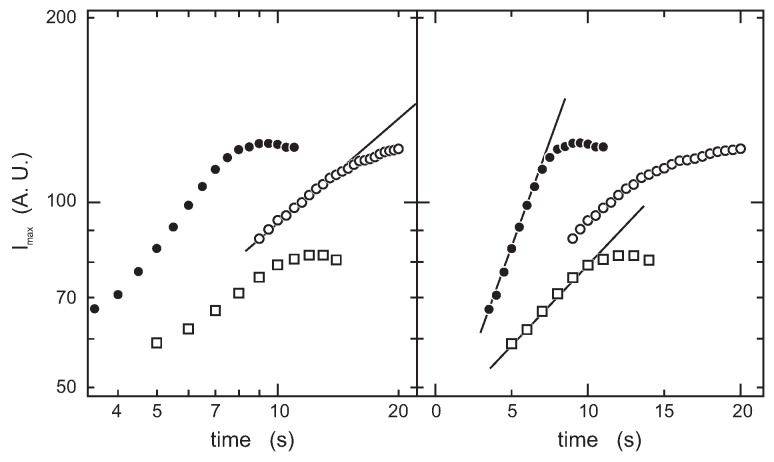
The time evolution of $I_{\max}$. Results are shown for the $\varphi_{g}=0.05$ system (open circles), the $\varphi_{g}=0.1$ system (closed circles) and the $\varphi_{g}=0.2$ system with single quench (open squares). The left figure is the double logarithmic plot of the results. The right figure represents the semi-logarithmic plot of the intensity results. The weight fraction of PEG-1000 is fixed at 0.1. The slope of the line drawn for the $\varphi_{g}=0.05$ system in the left figure is about 1.3.

The time evolution of the scattering light intensity from the system is also discussed together with the time evolution of $q_{\max}$ in the dynamics of the phase separation. The scattering light intensity at the position of the maximum, $I_{\max}$, is shown as a function of time, *t*, in the double and semilogarithmic plot in Figure 8. Two kinds of time evolution are observed in this system depending on the weight fraction of gelatin. The time evolution of the scattered light intensity in the $\varphi_{g}=0.05$ system in the time region around 10 s seems to be expressed by a power law relationship $I_{\max}\propto t^{\beta }$ with β=1.3, as shown in the double logarithmic plot in Figure 8. On the other hand, $I_{\max}$ increases with an exponential-like growth in the $\varphi_{g}=0.1$ and $\varphi_{g}=0.2$ systems, as shown in the semilogarithmic plot of Figure 8. Since the time interval of the measurement is limited, we do not strongly claim the above results. It is, however, worth noting here that the time evolution in the spinodal decomposition process is divided into an early and a late stage, and the scattering light intensity increases as a function of time with an exponential-like growth in the very early stage of decomposition. The time interval of the measurements in the $\varphi_{g}=0.1$ and $\varphi_{g}=0.2$ systems may correspond to the very early stage of the spinodal decomposition. The results may be natural since the gelation reaction accelerates the spinodal decomposition in these two systems. The power law relationship for the $\varphi_{g}=0.05$ system may also suggest the presence of the scaling relation, β≥3α [31]. The detailed discussion of these results requires further precise experimental studies of the system.

## 4. Conclusions

The phase behaviors of the ternary system of gelatin, water and PEG oligomer are studied as a function of the weight fraction of gelatin and the weight fraction and molecular weight of PEG oligomer. In addition to the sol-gel transition line, the phase separation line appears in the lower concentration region of the phase diagram when PEG oligomer coexists in the aqueous gelatin solution. The phase separation line is shifted to the higher temperature region upon increasing the concentration and/or the molecular weight of PEG oligomer. The phase behaviors of the system observed under present experimental conditions are well explainable by the site-bond correlated-percolation model of the polymer gelation. Since the phase separation line and the sol-gel transition line cross over in the phase space of $T-\varphi_{g}$, the phase space is typically divided into four regions. The regions thus appearing in the phase space correspond to the definite phases: one-phase sol, one-phase gel, two-phase sol and two-phase gel.

The transitions between these phases in a system with PEG-1000 at a weight fraction of 0.1 are studied by the light-scattering method. The light-scattering profile from the quenched sample solution shows a maximum at a certain magnitude of the scattering vector, $q_{\max}$. Both the time evolution of the magnitude of $q_{\max}$ and the scattering intensity at the maximum, $I_{\max}$, are analyzed.

The time evolutions of $q_{\max}$ and $I_{\max}$ for the system of $\varphi_{g}=0.05$ show the typical behaviors that correspond to the simple spinodal decomposition of the liquid-liquid phase separation process. The results are consistent with the previous optical microscope observations.

The similar behaviors of $q_{\max}$ and $I_{\max}$ in the $\varphi_{g}=0.05$ system are observed for the $\varphi_{g}=0.1$ system. The results suggest that the spinodal decomposition also plays a role in the phase transition of this system. However, $q_{\max}$ and $I_{\max}$ change much earlier and faster than in the $\varphi_{g}=0.05$ system, suggesting the acceleration of the spinodal decomposition. Two reasons are considered for the acceleration of the spinodal decomposition. One is the attractive interaction due to the gelation, and the other is the effects of the viscoelastic phase separation.

In contrast to the above two systems, the time evolution of $q_{\max}$ is completely altered from the simple spinodal decomposition in the single quench process of the $\varphi_{g}=0.2$ system. The results indicate that the sol-gel transition plays a dominant role in all of the transition behaviors. The stepwise quench experiments are done to separate the effects of the sol-gel transition and the phase separation. The results indicate that the phase separation occurs even in the polymer network of the gel. However, the phase separation is limited to a microscopic scale because of the presence of the polymer network of the gel. The results suggest that the phase separation line, when it appears in the gel phase, should be regarded as the microphase separation line.

## 5. Materials and Methods

Gelatin (Type-B, alkali-treated gelatin, No. 1040781000, Merck, Darmstadt, Germany, M. W. $≃6.9\times 10^{4}$) was obtained from Merck and used as obtained. The average molecular weight of gelatin was determined by gel permeation chromatography.

The oligomers of PEG, namely diethylene glycol (M. W. = 106, PEG-100), triethylene glycol (M. W. = 150, PEG-150) and tetraethylene glycol (M. W. = 194, PEG-200) were obtained from Wako Pure Chemical Industry (Osaka, Japan). The polymers of PEG at M. W. ∼ 400 (PEG-400) and PEG at M. W. ∼ 1000 (PEG-1000), were obtained from Nichiyu Corporation (Tokyo, Japan) and Sanyo Chemical Industry (Kyoto, Japan), respectively.These oligomers and polymers are also used as obtained.

Firstly, water and ethylene glycols were mixed at the desired ratio to make the mixed solvent of gelatin. After ethylene glycol is dissolved completely into water, the calculated amount of gelatin was added into the mixture of water and ethylene glycol. The solutions thus made are heated up to 60 ${}^{\circ }$C to dissolve the gelatin. The obtained solution was transferred into a temperature-controlled bath at a temperature of 60 ${}^{\circ }$C, and then, the temperature is lowered at a rate of 10 ${}^{\circ }$C/h. The solution became opaque near the phase separation temperature. The opaqueness of the solution near the phase separation temperature is caused by the structure change and the formation of the phase-separated droplets by the aggregation of polymers. It has been clarified that the cloud point temperature of the polymer solution practically coincides with the phase separation temperature within an accuracy of a few millidegrees [33,34]. Therefore, the cloud point temperature, which is determined by visual inspection, is assigned as the phase separation temperature in this study. The accuracy of the phase separation temperature thus determined was already confirmed by comparing the data with the light-scattering measurement and the turbidity measurement in previous studies [11,12]. The turbidity of the solution was also measured as a function of the temperature if necessary for the cross-check of the phase separation temperature. After the determination of the phase separation temperature, the solution is kept at 15 ${}^{\circ }$C for 12 h to ensure the gelation. The gelation temperature was determined in this heating process by the falling ball method using a Teflon ball with a wait of 16 mg. Firstly, the Teflon ball was placed on the surface of the gel. The heating rate was chosen as 5 ${}^{\circ }$C/h. The gelation temperature was determined as that at which the ball on the surface of the gel falls into the solution. In this method, the applied stress due to the ball is balanced with the elasticity of the polymer network of the gel. However, the elasticity due to the polymer network diminishes when the system attains the sol-gel transition temperature. The ball falls into the gel when the stress due to the ball overcomes the elasticity of the polymer network. Therefore, the gelation temperature that is determined by the falling ball method corresponds to the fracture temperature of the polymer network of the gel. Although the elastic modulus is not measured, the falling ball method is one of the rheological methods. The weight of the ball should be as small as possible to determine the correct gelation temperature.

It is well known that gelatin is easily degraded by the autocatalysis reaction in the solution state. Thus, it is not favorable to expose gelatin solution to a higher temperature. The cooling rate to determine the phase separation temperature was 10 ${}^{\circ }$C/h, taking into account that the degradation of gelatin should be avoided. Conversely, the slower heating rate was chosen for the exact determination of the gelation temperature by the falling ball method. The phase diagram obtained here, therefore, did not represent an equilibrium state since the cooling rate was rather high. It was, however, sufficient for the present purpose because the phase separation in the liquid state occurs rather quickly. In addition, the thermal hysteresis is not significant in the case of gelatin gel. Further details were given in the previous report [12].

The small angle laser light-scattering measurements were taken using a homemade apparatus. The details of the apparatus are given in [35]. Our apparatus consists of a He-Ne laser (JDSU Uniphase, Milpitas, CA, USA; 8 mW, λ = 632.8 nm) and the one-dimensional detector (Hamamatsu, Japan; S3901-512Q; 512 channels, 50 μm width/channel). The distance between the sample and the detector was changed from about 2 to 30 cm by a one-dimensional translator. Multiple scattering from the sample virtually dominates in the strongly-opalescent region. Such effects are crucial in the detailed discussion near the critical point. In the present system, however, the strong opalescence becomes dominant only in the late stage of the spinodal decomposition. For the present system, a suitable way of minimizing the effects of the multiple scattering is to suppress it by shortening the optical path length in the cell and to work at a larger wavelength because the scattering intensity is proportional to $\lambda^{-4}$. The sample gel was, therefore, prepared in the optical cell of a thickness from 1 to 10 mm, depending on the opacity of the gel. The scattering data were obtained by a computer system. The scattering profiles from the gels thus obtained are expressed as a function of the scattering vector, q=(4πn/λ)sin(θ/2), where *n*, θ and λ are the refractive index, the scattering angle and the wave length of the incident light [36].

## Abbreviations

The following abbreviations are used in this manuscript:

PEG	poly(ethylene glycol)
PEG-100	diethylene glycol
PEG-150	triethylene glycol
PEG-200	tetraethylene glycol
PEG-400	PEG at M. W. ∼400
PEG-1000	PEG at M. W. ∼1000
$q_{\max}$	magnitude of the scattering vector at the position of the maximum in the scattering profile
$I_{\max}$	scattering light intensity at the position of the maximum in the scattering profile
